# Drp1 regulates mitochondrial dysfunction and dysregulated metabolism in ischemic injury via Clec16a-, BAX-, and GSH- pathways

**DOI:** 10.1038/s41419-020-2461-9

**Published:** 2020-04-20

**Authors:** Chenyang Duan, Lei Kuang, Xinming Xiang, Jie Zhang, Yu Zhu, Yue Wu, Qingguang Yan, Liangming Liu, Tao Li

**Affiliations:** State Key Laboratory of Trauma, Burns and Combined Injury, Second Department of Research Institute of Surgery, Daping Hospital, Army Medical University, 400042 Chongqing, P. R. China

**Keywords:** Apoptosis, Autophagy, Mitochondria, Metabolic disorders, Trauma

## Abstract

The adaptation of mitochondrial homeostasis to ischemic injury is not fully understood. Here, we studied the role of dynamin-related protein 1 (Drp1) in this process. We found that mitochondrial morphology was altered in the early stage of ischemic injury while mitochondrial dysfunction occurred in the late stage of ischemia. Drp1 appeared to inhibit mitophagy by upregulating mito-Clec16a, which suppressed mito-Parkin recruitment and subsequently impaired the formation of autophagosomes in vascular tissues after ischemic injury. Moreover, ischemia-induced Drp1 activation enhanced apoptosis through inducing mitochondrial translocation of BAX and thereby increasing release of Cytochrome C to activate caspase-3/-9 signalling. Furthermore, Drp1 mediated metabolic disorders and inhibited the levels of mitochondrial glutathione to impair free radical scavenging, leading to further increases in ROS and the exacerbation of mitochondrial dysfunction after ischemic injury. Together, our data suggest a critical role for Drp1 in ischemic injury.

## Introduction

Ischemia, a condition marked by insufficient blood supply to tissues^1^, hallmarks haemorrhagic shock^2^, myocardial infarction, stroke^3^, and tumorigenesis^4^. The extent of tissue injury is associated with the extent of oxygen deprivation and the duration of the ischemic period^5^. In the early stages of ischemia, vasoconstriction in the skin and viscera is prominent due to activity in the sympathetic-adrenal medullary system, which compensates for the decrease in blood pressure to maintain adequate blood flow to the heart and brain^6^. In the late-stage ischemia, vascular hyporeactivity with a progressive decrease in blood pressure may cause multiple organ dysfunction, and even death^7^.

Mitochondria are a major target in hypoxic/ischaemic injury, while increasing mitochondrial damage occurs with prolonged ischemic duration^8^, which in turn compromises cellular bioenergetics^9^. However, whether the perturbation of mitochondrial homeostasis and cellular energetics in the vascular system occurs in a time-dependent manner after ischemic injury remains unknown.

The balance of mitochondrial dynamics, including mitochondrial fission and fusion, ensures the maintenance of mitochondrial function under physiological conditions^10^. Mitochondrial morphology is governed by the balance of mitochondrial fusion, mediated by mitofusins and optic atrophy 1 (OPA1), and fission, mediated by dynamin-related protein 1 (Drp1). Disordered mitochondrial dynamics alter metabolism, proliferation, apoptosis and mitophagy, all contributing to serious diseases including neurodegenerative syndromes, pulmonary arterial hypertension (PAH), and cancer^11–15^. Ischaemia may cause vascular dysfunction through Drp1-mediated mitochondrial fission^16^. Recent studies have shed light on the roles of Drp1 in ischemia-induced cellular dysfunction in traumatic injury^10^. However, a comprehensive and systematic study has yet to be conducted on the specific regulatory mechanism of Drp1 on homeostasis of the internal environment of mitochondria after ischemic injury.

Here, we used microarray data from rats in different phases of ischaemic injury to study the effects of varying degrees of ischaemic injury on the molecular pathways linked to mitochondria. In addition, we used Drp1 knockout (KO) mice (Drp1±) to determine the function of Drp1 in biomedical processes under physiological and ischaemic conditions.

## Materials and methods

### Animals

Sprague-Dawley (SD) rats were purchased from Army Medical University (Chongqing, China) and Drp1 KO mice (Drp1±) were generated by Shanghai Model Organisms Center, Inc. (Shanghai, CHINA). The Mouse Genome Informatics (MGI) database (http://www.informatics.jax.org) showed that mice homozygous for the knockout Drp1 allele exhibit embryonic lethality. The frameshift and functional loss of the Drp1 gene reading frame was achieved by CRISPR/Cas9 and non-homologous recombination repair to pull in the mutation and obtain Cas9 mRNA/gRNA. F0 Drp1 KO mice generation was obtained afterwards. The transcript Ensembl for Drp1 KO was Dnm1l-203 (ENSMUST00000115749.2) and the targeted exon was exon 2^17^. All procedures were approved by the Research Council and Animal Care and Use Committee of the Research Institute of Surgery, Army Medical University (Chongqing, China). The investigation conformed to the protocols in the Guide for the Care and Use of Laboratory Animals (National Institutes of Health, Publication No. 85–23, Revised 1996) and single bind method for analysis was applied. Randomization was used to group the animals.

### Materials

Antibodies for Drp1 (ab56788), GDAP1 (ab100905), Tomm20 (ab186734), Pi3kca (ab40776), Hif1a (ab51608), Bik (ab41716), Amd1 (ab127576), Pink1 (ab23707), Parkin (ab77924), Bnip3 (ab219609), Bnip3l (ab155010), mTOR (ab32028), Phospho-mTOR (ab109268), LC3 (ab192890), Clec16a (ab75346), Lamp1 (ab24170), BAX (ab32503), CytC (ab133504), Caspase-3 (ab13847), Caspase-9 (ab32539), β-actin (ab8226), and COX4 (ab110272) were purchased from Abcam (Cambridge, MA, USA). Antibodies for Drp1 Ser616 (CST#3455) and Ser637 (CST#4867) were purchased from Cell Signaling Technology (Danvers, MA, USA). Antibodies for Pdzd8 (orb1959), Inf2 (orb309456) were purchased from Biorbyt (Cambridge, UK). MitoTracker Deep Red (M22426) was purchased from Invitrogen (Carlsbad, CA, USA). The JC-1 fluorescent probes for mitochondrial membrane potential (△Ψm) detection (C2006), Calcein probes for mitochondrial permeability transition pore (mPTP) opening detection (C2009S), DCFH-DA fluorescent probes for reactive oxygen species (ROS) detection (S0033), glutathione (GSH) and oxidized glutathione (GSSG) Assay Kit (S0053), Ad-mCherry-GFP-LC3B (C3011) and GFP-LC3 (C3006) were purchased from Beyotime Biotechnology (Shanghai, CHINA). In situ Cell Death Detection Kit (TUNEL) (11767291910) was purchased from Roche (Basel, Switzerland). LysoTracker Deep Red (L7528) was purchased from Thermo Scientific (Waltham, MA, USA). The Mitophagy detection kit (MD01) was purchased from Dojindo Molecular Technology (Tokyo, JAPAN). Protein A/G magnetic beads for immunoprecipitation (MB11250) was purchased from Sino Biological (Houston, Texas, USA).

The vascular smooth muscle cell line (VSMC) was obtained from the Cell Bank of the Chinese Academy of Sciences (Shanghai, CHINA). Drp1 shRNA (short hairpin RNA) was generated by Obio Technology (Shanghai, CHINA). Primers for Drp1 KO genotype were Forward-GTGCCACTCGGACTGCCTTCT and Reverse-GACCTGCTCCCCACATCAACA while primers for WT genotype were Forward- GTGCCACTCGGACTGCCTTCT and Reverse- CACTGAGCTCTTCCCACTGC (KO = 1115 bp and WT = 2242 bp). All other chemicals were purchased from Sigma unless specifically mentioned otherwise.

### Model preparation

To prepare the ischemia model, rats (220–240 g) were anaesthetized with sodium pentobarbital (initial dosage, 30 mg/kg). Anaesthetized rats were placed on a warmed plate to maintain body temperature at 37 °C. Aseptic techniques were adopted for all surgical procedures. The right femoral arteries were catheterized with polyethylene catheters for bleeding 50% of total blood volume (≈7% of weight). Measurement of ischemia time was started after the model was established^18^. At the end of ischemia, rats were sacrificed with a lethal dose of sodium pentobarbital (100 mg/kg, iv). A laparotomy was then carried out to obtain superior mesenteric artery tissue (SMAs). The same procedure was also carried out on Drp1 KO mice (20–25 g).

For hypoxia treatment, VSMCs were placed in a hypoxia compartment and bubbled with hypoxic gas (95% N_2_ and 5% CO_2_) at 3 L/min for 15 min, followed by a 10-min rest. This procedure was repeated five times until the O_2_ concentration was below 0.2%. The cells were then cultured in hypoxic conditions and maintained for 1 h or 4 h, corresponding to the ischemia conditions in vivo^19^.

### Gene expression profiling with Agilent microarrays

Total RNA was extracted from isolated SMAs by Trizol^®^ (Invitrogen, CA, USA) followed by purification using RNeasy columns (Qiagen, Hilden, Germany) according to protocol. The quantity and quality of RNA preparations were evaluated on an Agilent 2100 Bioanalyzer with RNA6000 Nano Reagents and Supplies (Agilent, Santa Clara, CA, USA). Quality-checked RNAs were then transcribed with the First-Strand cDNA Synthesis Kit (Agilent, Santa Clara, CA, USA); the original microarray data from this study are available at the NCBI GEO database (http://www.ncbi.nlm.nih.gov/geo/). The 9 SD rat chips of 3 ischemic conditions (Normal, Ischemia 1 h, Ischemia 4 h) from three individuals are available under the accession number GSE123542. For the database of Drp1-knockout mice, 14 chips from Drp1 +/+ and Drp1 +/− mice (Normal_Drp1 +/+: 3 individuals, Normal_Drp1 +/−: 4 individuals, Ischemia_ Drp1 +/+: 3 individuals, Ischemia_Drp1 +/−: 4 individuals) are available under the accession number GSE124096.

### Analysis of microarray data

The normalized ratio of gene expression signals was log2 transformed, and hierarchical clustering was performed with average linkage. Paired-end analysis was used to locate segments in sequence assembly and pacBioToCA genome assembly was used to map the data. The clustered heatmap was visualized using Treeview. The RVM (Random variance model) F-test was applied to filter differentially expressed genes (DEGs) for the different situations. After the significance analysis and FDR (false discovery rate) analysis, we selected the differentially expressed genes according to an FDR threshold set at FDR < 0.05, and according to the fold changes of any two groups >1.5.

A Series-Cluster analysis was performed to identify the global trends of mitochondrial DEGs in relation to either time after ischemia or time after Drp1 knockdown. Fisher’s exact test was applied to identify the model profiles with probability significantly higher than expected at random^20^.

Gene ontology (GO) analysis^21^ was performed to help elucidate the biological process (BP), molecular function (MF), and cellular component (CC) of unique genes in either the significant or representative profiles. GO analysis was conducted to identify the main function of the genes having the same expression trend according to GO analysis. Fisher’s exact test and χ^2^ test were applied to identify the significant GO categories, and FDR was used to correct p-values.

### Analysis of metabolic profiling

Principal Component Analysis (PCA)^22^ was performed to orthogonally transform a set of observed variables into linear data in order to reveal the internal structure of the data. Abscissa PC1 and ordinate PC2 in the PCA score scatter plot represent the scores of the first and second principal components, respectively. Multivariate Analysis was used to assess the relationship between metabolites and their promotion or antagonism of biological processes. The screening criteria for differential metabolites were: Variable Importance in the Projection (VIP) value >1 and *p*-value for Student’s *t*-test <0.05. Volcano plots were produced to visualize the results of screening differential metabolites. The results of the metabolic pathway analysis were displayed by bubble diagram. Each bubble in the bubble diagram represents a metabolic pathway. The abscissa and size of the bubble indicated the size of the pathway’s influence factor in the topological analysis. The larger the size of the bubble, the greater the pathway influence. The longitudinal coordinate and colour of the bubble represent the *p*-value of the enrichment analysis (- lnP-value). The smaller the *p* value, the more significant the degree of pathway enrichment.

### Twenty-four hour metabolic cage detection

The metabolic cage was preheated for 30 min and loaded with the same weight of feed and drinking water. After gas calibration, the mice were weighed and placed in the corresponding metabolic cage. The Oxymax software and default hardware configuration files were used to continuously measure the oxygen consumption (VO2), respiratory quotient and heat levels of each individual for 24 h.

### Subcellular fractionation

Isolated SMAs or VSMCs were collected in filter cartridges. Cytosol fractions were isolated using a MinuteTM Cytoplasmic Extraction Kit (Invent Biotechnologies, Inc. SC-003, Beijing, CHINA). Mitochondria fractions were isolated using a MinuteTM Mitochondria Isolation Kit (Invent Biotechnologies, Inc. MP-007, Beijing, CHINA)^23^. Fractioned proteins were used for immunoblotting analyses with the indicated antibodies.

### Confocal microscopy observation

For confocal imaging, the Leica TCS SP5 (Leica Microsystems, Wetzlar, Germany) inverted confocal microscope was used. VSMCs were seeded in confocal culture plates at a density of 1 × 10^5^ cells per well. VSMCs were transfected with Ad-mcherry-GFP-LC3B for 24 h and then incubated in 10% FBS complete medium for another 24 h after removing the virus solution. Mitochondria were incubated with MitoTracker Deep Red (100 nM for 30 min, 37 °C), which was excited by 633 nm laser and emission was collected at 558–617 nm^24^. Cellular lysosomes were incubated with LysoTracker Deep Red (100 nM for 30 min, 37 °C), which was excited by 647 nm laser and emission was collected at 668 nm^25^.

### Transmission electronic microscopy imaging

Fresh SMA tissues were quickly fixed with arsenate buffer containing 2.5% glutaraldehyde for 24 h (pH = 7.4, 4 °C). After three 10 min-washes with 0.13 M Phosphate Buffered Saline (PBS), the tissues were post-fixed in 1% OsO_4_ for 2 h at room temperature and then dehydrated in a graded series of ethanol (65%, 70%, 75%, 80%, and 95% for 10 min each). Subsequently, the tissues were incubated with tert-butoxide for 10 min and then dried with CO_2_ (carbon dioxide), stained with uranyl acetate or lead citrate, and coated with gold (Au) using ion sputter coater. Finally, samples were viewed and imaged with a transmission electron microscope (H-7500, Hitachi Company, Japan)^26^.

### mPTP opening detection

VSMCs were seeded in 20 mm-diameter confocal petri dishes at a density of 1 × 10^5^ cells per well. mPTP opening detection was based on the protocol described previously^27^. Hypoxia-treated VSMCs were co-incubated with Calcein (2 μM) and MitoTracker (100 nM) for 30 min. After washing twice with PBS, the cells were then exposed to CoCl_2_ (2 mM) for 15 min to detect the distribution of cobalt inside mitochondria. The degree of mPTP opening was reflected by Red (MitoTracker)/Green (Calcein) fluorescence.

### Mitophagy assay

VSMCs were seeded in 20-mm-diameter confocal petri dishes at a density of 5 × 10^4^ cells per well. Mitophagy in live cells was monitored using the Mitophagy detection kit (Dojindo Molecular Technologies)^28^. The level of mitophagy was defined by the area of Mtphagy dye per cell. At least 50 cells were qualified in each group. The levels of colocalization of both Mtphagy dye and lysosome dye were also analysed. Quantification analysis was carried out using Image J.

### TUNEL assay

VSMCs were incubated on 20-mm-diameter petri dishes at a density of 5 × 10^4^ cells and fixed with 4% paraformaldehyde at room temperature for 60 min. After washing three times with PBS for 5 min, cells were incubated with 0.1% Triton-100 PBS for 5 min in an ice bath. The TUNEL detection solutions were prepared as previously described^29^ and 50 ul were added into each petri dish. After incubation, the solutions were then washed three times (5 min each) and DAPI was added to stain nuclei. The TUNEL florescent probe was excited by a 488 nm laser and the detection wavelength was set from 515 to 565 nm. Quantification for the TUNEL assay was conducted using Image J to measure the FITC fluorescence intensity.

### ROS and mitochondrial membrane potential (△Ψm) assay

VSMCs were seeded in 20-mm-diameter confocal petri dishes at a density of 5 × 10^4^ cells per well. After hypoxia treatment, the cells were stained with 1% DCFH-DA ROS fluorescent probes or 0.1% JC-1 fluorescent probes and incubated for 20 min at 37 °C. The ROS fluorescence and JC-1 monomer were excited by a 488 nm laser and emission was collected at 501–563 nm. The JC-1 aggregate fluorescence was excited by a 633 nm laser and emission was collected at 558–617 nm. Quantification for the ROS assay was conducted using Image J to measure the FITC fluorescence intensity, and quantification for the △Ψm assay was carried out to measure the fluorescence intensity ratio of JC-1 aggregate/JC-1 monomer.

### GSH and GSSG measurements

Vascular tissues were ground into powder with liquid nitrogen and treated with compounds according to the instructions. After the samples were harvested, lysed and centrifuged, the supernatant was used to determine mitochondrial glutathione (GSH) and oxidized glutathione (GSSG) levels using a GSH and GSSG Assay Kit by an Infinite M200 Pro microplate reader (Tecan, USA). GSH levels and the GSH/GSSG ratio were calculated as previously described^30^.

### Co-Immunoprecipitation (Co-IP)

Co-Immunoprecipitation (Co-IP) was performed using the Protein A/G Magnetic Beads IP Kit according to the manufacturer’s instructions. Ten microgram BAX antibody was diluted with 200 μl PBST in a tube. 50 μl Protein A/G Magnetic Beads was added into the mixture and then incubated for 10 min at room temperature, and the BAX-conjugated immunomagnetic beads were prepared after removing the supernatant on the magnetic separator. After the samples were harvested, lysed and centrifuged, the supernatants were gently mixed with BAX-conjugated immunomagnetic beads to prepare an immunomagnetic beads-antibody-antigen complex. After washing the beads with PBS three times, the above complex was resuspended in 100 μl PBS and was used to detect endogenous interaction between BAX and Drp1 by visualizing the intensity of Drp1 bands.

### Western blotting (WB)

Cell pellets were solubilized with RIPA buffer (Radio-Immunoprecipitation Assay, Beyotime Biotech, CHINA) with the addition of complete Protease Inhibitors (Roche, Switzerland) and Phosphatase Inhibitors (Roche, Switzerland), electrophoresed, and blotted onto PVDF (Polyvinylidene Fluoride) membranes. The membranes were incubated with indicated primary antibodies followed by incubation with horseradish peroxidase (HRP)-conjugated secondary antibodies (Beyotime Biotech, CHINA). Protein concentration was calculated by the BCA (Bicinchoninic Acid) Protein assay kit (Thermo Scientific Pierce, UK). Blotted proteins were visualized using an enhanced chemiluminescence detection kit (Tiangen Biotech, CHINA). The intensity of the bands was analysed by Quantity One V 4.62 Software (Bio-Rad, Life Science, USA). The relative protein expression was calculated by Image J software using the formula”Gray value of targeted protein band/Gray value of interior reference band (such as β-actin, COX4, etc)”.

### Statistics analysis

Results were expressed as means ± standard deviations (SD). Power test was used to estimate the sample size. One-way analysis of variance (ANOVA) was used for experiments with more than two groups and followed by Tukey’s post-hoc analysis. The survival analysis was calculated by the Kaplan-Meier method using SPSS (SPSS Inc., Chicago, IL, USA). *p* < 0.05 was considered statistically significant.

## Results

### Mitochondrial dysfunction is closely related to autophagy, apoptosis, and metabolism after ischemic injury

**Fig. 1:**
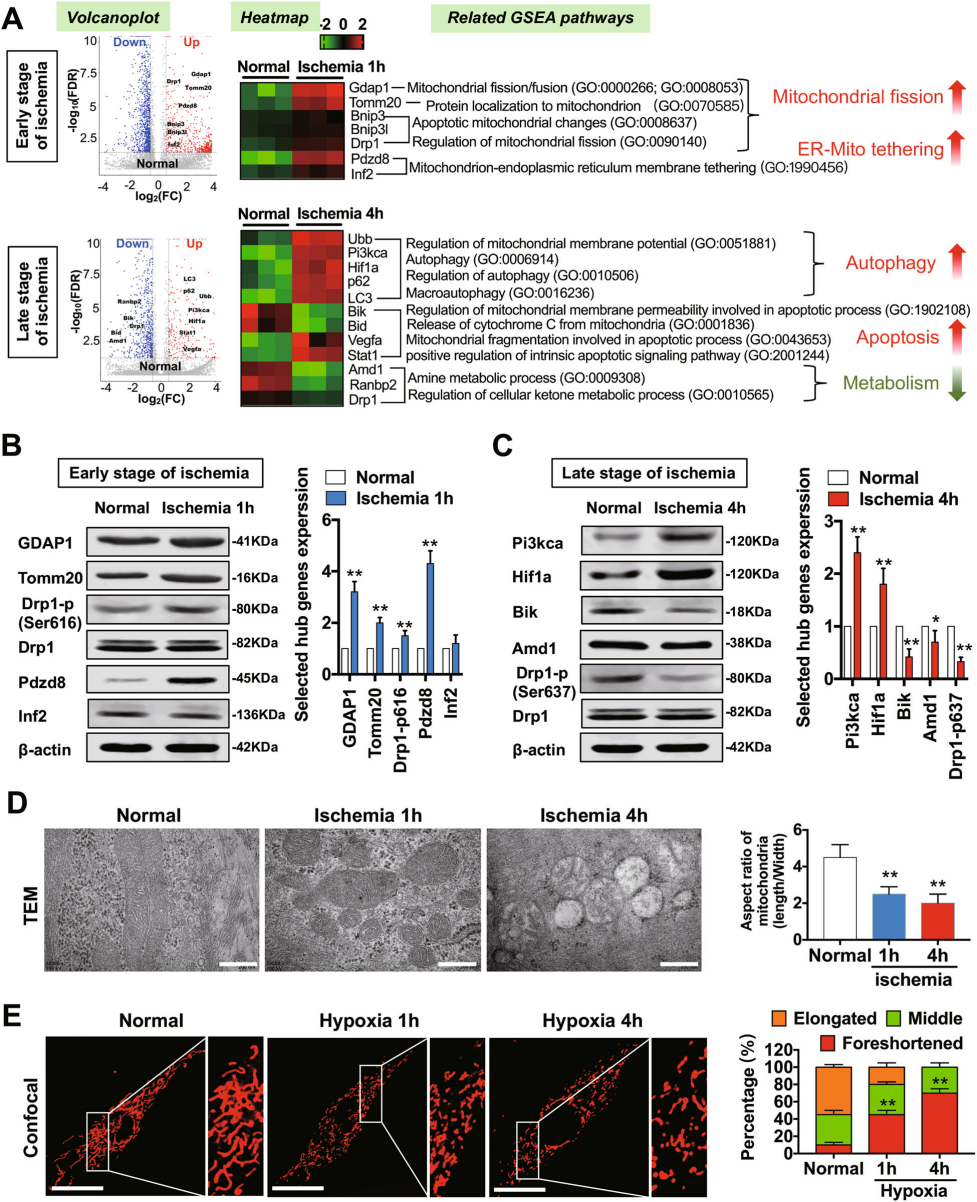
Molecular pathways linked to mitochondria in different phases of ischemia. **a** Mitochondrial-DEGs at 1 h and 4 h ischemic periods based on mitochondrial-genome microarrays. FDR values are shown in volcano plots and relative expression of DEGs for each sample are shown in heatmaps. The related GSEA pathways and GO ID numbers are listed. **b** Western blot and statistical analysis of representative hub genes for the 1 h ischemic period. **c** Western blot and statistical analysis of representative hub genes for the 4 h ischemic period. **d** TEM (transmission electronic microscopy) images to observe mitochondrial morphology of SMAs at 1 h and 4 h ischemic periods. (bar, 400 nm). **e** Confocal images to observe mitochondrial morphology of VSMCs in hypoxic conditions for 1 h and 4 h (63X_bar, 25 μm). Quantitation was performed in triplicate and scored into three categories: foreshortened, middle, and elongated mitochondria, with 100 cells scored per group. *p < 0.05 and **p < 0.01 compared to the normal group.

In order to investigate the dynamic changes in mitochondrial genomics after ischemic injury, we collected vascular tissue [superior mesenteric artery (SMA)] samples from rats at different durations of ischemia to assemble gene chips and conduct whole transcriptome sequencing. After detecting 1200 differential genes in 46,095 gene chip probes, we focused on mitochondria-related genes. The results showed that, in contrast to the normal group, the significantly altered mitochondria-related genes after 1-h ischemic injury included Gdap1, Tomm20, Bnip3, Bnip3l, Drp1, Pdzd8, and Inf2. GSEA revealed that these genes are mainly involved in the morphological changes of mitochondria (mainly the mitochondrial fission process) and the endoplasmic reticulum (ER)-mitochondria (Mito) tethering process. The significantly altered mitochondria-related genes after 4-h ischemic injury included Ubb, Pi3kca, Hif1a, p62, LC3, Bik, Bid, Vegfa, Stat1, Amd1, Ranbp2, and Drp1. The functional pathways involved included: (1) autophagy pathways such as mitochondrial membrane potential (△Ψm), mitophagy, and macroautophagy; (2) apoptosis pathways such as mitochondrial membrane permeability, Cytochrome C release, and apoptosis of innate cells; and (3) metabolic pathways such as amine metabolism and ketone metabolism (Fig. 1A).

Protein levels of several genes were assessed to verify the transcriptome results, showing that the expression levels of Gdap1, Tomm20, Pdzd8, and Drp1 Ser616 were clearly upregulated in the early stages of ischemic injury (1 h) (Fig. 1B); Pi3kca and Hif1a were upregulated while Bik, Amd1, and Drp1 Ser637 were notably downregulated in the late stages of ischemic injury (4 h) (Fig. 1C).

To verify the functional pathways of mitochondria-related genes by GSEA, we examined the morphologic changes of mitochondria in vascular tissues after ischemic injury with a transmission electron microscope (TEM) and found that the number of mitochondria increased substantially in the early stage of ischemia, and that the aspect ratio of mitochondria decreased by 50% (Fig. 1D). We further simulated ischemic injury at the cellular level via hypoxia treatment of vascular smooth muscle cells (VSMCs). The mitochondria labelled by MitoTracker^®^ were mostly in elongated or thread shapes under normal conditions, while mitochondrial fragmentization became more pronounced and the quantity of foreshortened mitochondria increased 4-fold after hypoxia treatment (Fig. 1E). These results suggest that mitochondrial morphology changed in the early stage of ischemic injury and manifested primarily as the occurrence of excessive mitochondrial fission.

**Fig. 2:**
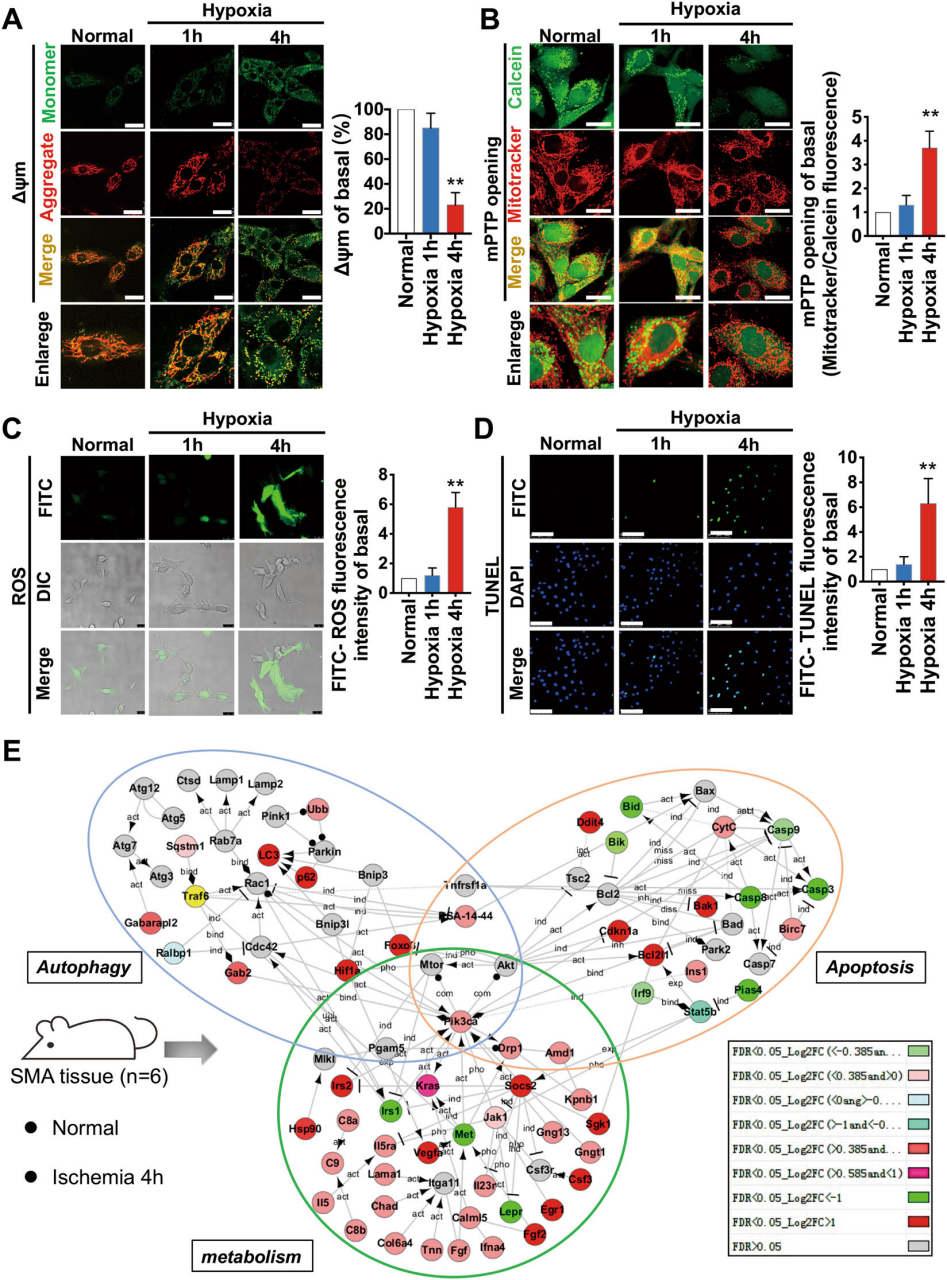
Mitochondrial dysfunction is closely related to the processes of autophagy, apoptosis, and metabolism after hypoxia in VSMCs. **a** Representative confocal images and statistical analysis of mitochondrial transmembrane potential (Δψm) which were labelled by JC-1 monomer (green fluorescent probe) and JC-1 aggregate (red fluorescent probe) in VSMCs in hypoxic conditions for 1 h and 4 h (40X_bar, 25 μm). **b** Representative confocal images and statistical analysis of mPTP openings, which were labelled by calcein (green fluorescent probe) and MitoTracker® (red) in VSMCs in hypoxic conditions for 1 h and 4 h (40X_bar, 25 μm). **c** Representative confocal images and statistical analysis of ROS generation in VSMCs in hypoxic conditions for 1 h and 4 h (40X_bar, 25 μm). **d** Representative confocal images and statistical analysis of TUNEL results in hypoxic conditions for 1 h and 4 h (20X_bar, 100 μm). **e** Co-expression network between gene clusters associated with Autophagy, Apoptosis, Metabolism, and screened mitochondria-related gene clusters. *p < 0.05 and **p < 0.01 compared to the normal group.

Next, we compared the TEM results for mitochondria in 1 h- and 4 h- ischemic conditions (Fig. 1D) and found that in the late stage of ischemia (4 h), the internal structure of mitochondria was disorganized and the vacuolization was severe. To examine whether mitochondrial dysfunction may occur in the late stage of ischaemic injury, we studied the variations in mitochondrial function after hypoxia treatment in VSMCs at different time points. We found that certain indices of mitochondrial function such as △Ψm, mPTP opening, ROS level, and TUNEL apoptosis registered no statistical difference compared to those in the normal group (*p* > 0.05) after 1-h hypoxia treatment, while after 4-h hypoxia treatment, △Ψm decreased by 77% (Fig. 2A), mPTP opening increased 2.7-fold (Fig. 2B), ROS level was upregulated 4.8-fold (Fig. 2C), and the FITC value of the TUNEL index increased 5.3-fold (Fig. 2D) (*p* < 0.05). These results suggest that mitochondrial dysfunction may occur in the late stage of ischemic injury, consistent with GSEA results. We further screened the gene clusters associated with autophagy, apoptosis, and metabolism in pre-transcriptome differential genes and constructed a dynamic co-expression network (Fig. 2E) of mitochondria-related gene clusters screened from Fig. 1A, from which we found that essential genes such as Drp1, Pik3ca, and Hif1a may play roles in regulating multiple functional pathways after ischemic injury.

### Drp1 plays an important role in the regulation of mitochondrial and other essential functional pathways after ischemic injury

To study the function of Drp1 under physiological and ischemic injury conditions, we conducted whole-genome sequencing with Drp1 KO mice (Fig. S1A,B). The sequencing results showed that the gene read value of exon 2 in Drp1 KO mice was distinctly lower than that of WT (Fig. S1,C), while western blot results also showed that the protein expression of Drp1 decreased by approximately 40% after Drp1 KO (Fig. S1,D), both of which verified the knockout effect. Meanwhile, immunofluorescence images displayed lower Drp1 expression in the SMA of Drp1 KO mice compared with that in WT under both normal and ischemic injury conditions (Fig. S1,E).

**Fig. 3:**
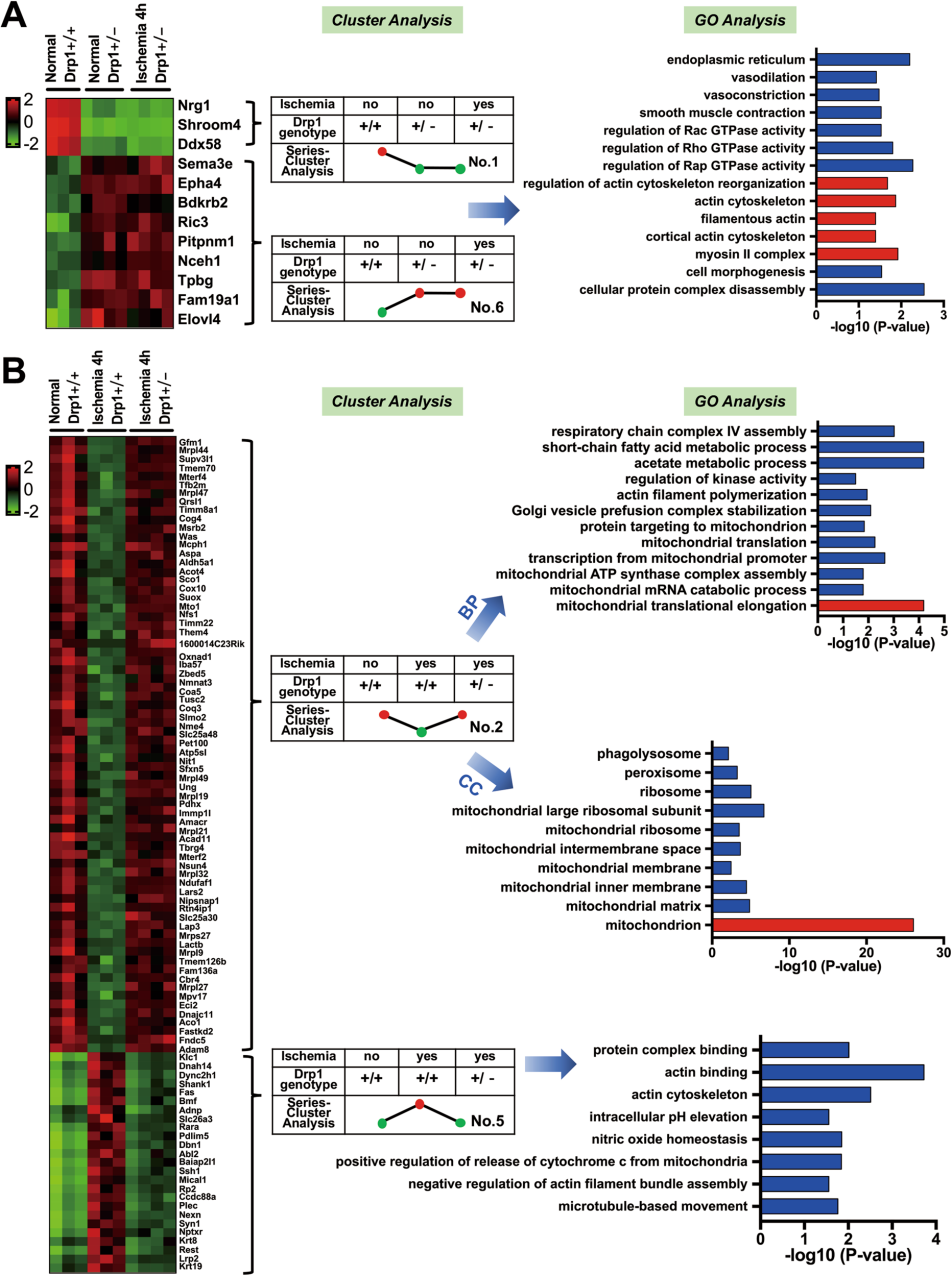
The functional and pathway enrichment analysis for Drp1-mediated biological pathways under normal and ischemic conditions. **a** Functional enrichment analysis for Drp1-mediated biological pathways under physiological conditions. **b** Functional enrichment analysis for Drp1-mediated biological pathways under ischemic conditions. GO analysis results are listed after selected trends of series-cluster data.

To detect the regulatory pathways mediated by Drp1 under physiological conditions, we first conducted series-cluster analysis on three groups [“Normal_Drp1 +/+”, “Normal_Drp1 +/−”, and “Ischemia 4h_Drp1 +/−” (data from 11 samples in total)] and then selected the trend analysis results of No. 1 and No. 6 (differential gene clusters under normal conditions after Drp1 KO but no obvious change after ischemic injury) to reflect Drp1-mediated functional pathways under physiological conditions (Fig. S2A). The functional and pathway enrichment analysis showed that Drp1 may be involved in the following biological processes under physiological conditions: (1) regulation of the GTPase activity of Rac, Rho, Rap, etc. (2) regulation of protein complex assembly, myosin II complex formation, actin cytoskeleton regulation, etc. (3) regulation of endoplasmic reticulum morphology and its functions; and (4) regulation of vasoconstriction or vasodilation (Fig. 3A).

Next, we conducted series-cluster analysis on another three groups [“Normal_Drp1 +/+”, “Ischemia 4h_Drp1 +/+”, and “Ischemia 4h_Drp1 +/−” and then selected the trend analysis results of No. 2 and No. 5 to reflect the Drp1-mediated functional pathways in ischemic injury conditions (Fig. S2,B). The No. 2 trend analysis enriched 12 GO_BP (Biological Process) pathways and 10 GO_CC (Cell Component) pathways. The No. 5 trend analysis enriched 5 GO_BP pathways, 1 GO_CC pathway, and 2 GO_MF (Molecular Function) pathways (Table 1). The enrichment analysis suggests that Drp1 might participate in the biological processes listed in Fig. 3B, such as actin filament aggregation, microtubule mobility, Cytochrome C release and NO homeostasis, etc, after ischemic injury.

**Table 1:** Functional enrichment analysis of Drp1-controlled potential pathways after ischemic injury.

	GO ID	GO Term	Different gene	*P*-Value
Down-up#2 GO_BP	GO:0070125	mitochondrial translational elongation	GFM1, MRPL44	6.46837E-05
	GO:0000958	mitochondrial mRNA catabolic process	SUPV3L1	0.016065818
	GO:0033615	mitochondrial proton-transporting ATP synthase complex assembly	TMEM70	0.016065818
	GO:0006390	transcription from mitochondrial promoter	MTERF4, TFB2M	0.002243482
	GO:0032543	mitochondrial translation	MRPL47, QRSL1	0.005522714
	GO:0006626	protein targeting to mitochondrion	MTERF4, TIMM8A1	0.014642313
	GO:0048213	Golgi vesicle prefusion complex stabilization	COG4	0.008065251
	GO:0030041	actin filament polymerization	MSRB2, WAS	0.011171288
	GO:0043549	regulation of kinase activity	MCPH1	0.031874961
	GO:0006083	acetate metabolic process	ASPA, ALDH5A1	6.46837E-05
	GO:0046459	short-chain fatty acid metabolic process	ALDH5A1, ACOT4	6.46837E-05
	GO:0008535	respiratory chain complex IV assembly	SCO1, COX10	0.000949805
Down-up#2 GO_CC	GO:0005739	mitochondrion	GFM1, SUOX, MTO1, NFS1, TIMM8A1, TIMM22, et al. (Counts:63 genes)	8.71271E-27
	GO:0005759	mitochondrial matrix	TFB2M, IBA57, COQ3, PDHX, et al. (Counts:9 genes)	1.44154E-05
	GO:0005743	mitochondrial inner membrane	THEM4, TIMM22, TIMM8A1, SCO1, et al. (Counts:12 genes)	3.67736E-05
	GO:0031966	mitochondrial membrane	COX10, ACAD11, SFXN5, TMEM126B	0.003633817
	GO:0005758	mitochondrial intermembrane space	THEM4, SUOX, SLMO2, NME4, TIMM8A1	0.000225776
	GO:0005761	mitochondrial ribosome	MRPL49, MRPL19, MRPL47	0.000325395
	GO:0005762	mitochondrial large ribosomal subunit	MTERF4, MRPL49, NSUN4, MRPL32, MRPL47, MRPL27	1.95634E-07
	GO:0005840	ribosome	MRPL47, MRPL49, MRPL19, MRPL21, et al. (Counts:10 genes)	9.65376E-06
	GO:0005777	peroxisome	FNDC5, AMACR, ACAD11, ACOT4, MPV17, ECI2	0.000568133
	GO:0032010	phagolysosome	ADAM8	0.008088235
Up-down#5 GO_BP	GO:0007018	microtubule-based movement	KLC1, DNAH14, DYNC2H1	0.017271489
	GO:0032232	negative regulation of actin filament bundle assembly	SHANK1	0.027955161
	GO:0090200	positive regulation of release of cytochrome c from mitochondria	FAS, BMF	0.014414304
	GO:0033484	nitric oxide homeostasis	ADNP	0.014076018
	GO:0051454	intracellular pH elevation	SLC26A3	0.027955161
Up-down#5 GO_CC	GO:0015629	actin cytoskeleton	RARA, PDLIM5, BMF, DBN1, ABL2, BAIAP2L1	0.003093628
Up-down#5 GO_MF	GO:0003779	actin binding	SSH1, MICAL1, DBN1, RP2, et al. (Counts:10 genes)	0.000189567
	GO:0032403	protein complex binding	FAS, NPTXR, KRT8, SHANK1, REST, LRP2, KRT19	0.009697945

The results reported above verified the close relationship between mitochondrial functions and functional processes including autophagy, apoptosis, and metabolism, as well as the key role of Drp1 in mitochondrial function regulation after ischemic injury. Whether Drp1 regulates the biological processes of autophagy, apoptosis, and metabolism directly after ischemic injury, as well as the exact mechanisms by which it exerts this regulatory role, remain unknown.

### Drp1 participates in autophagy after ischemic injury through the Clec16a-Parkin pathway

**Fig. 4:**
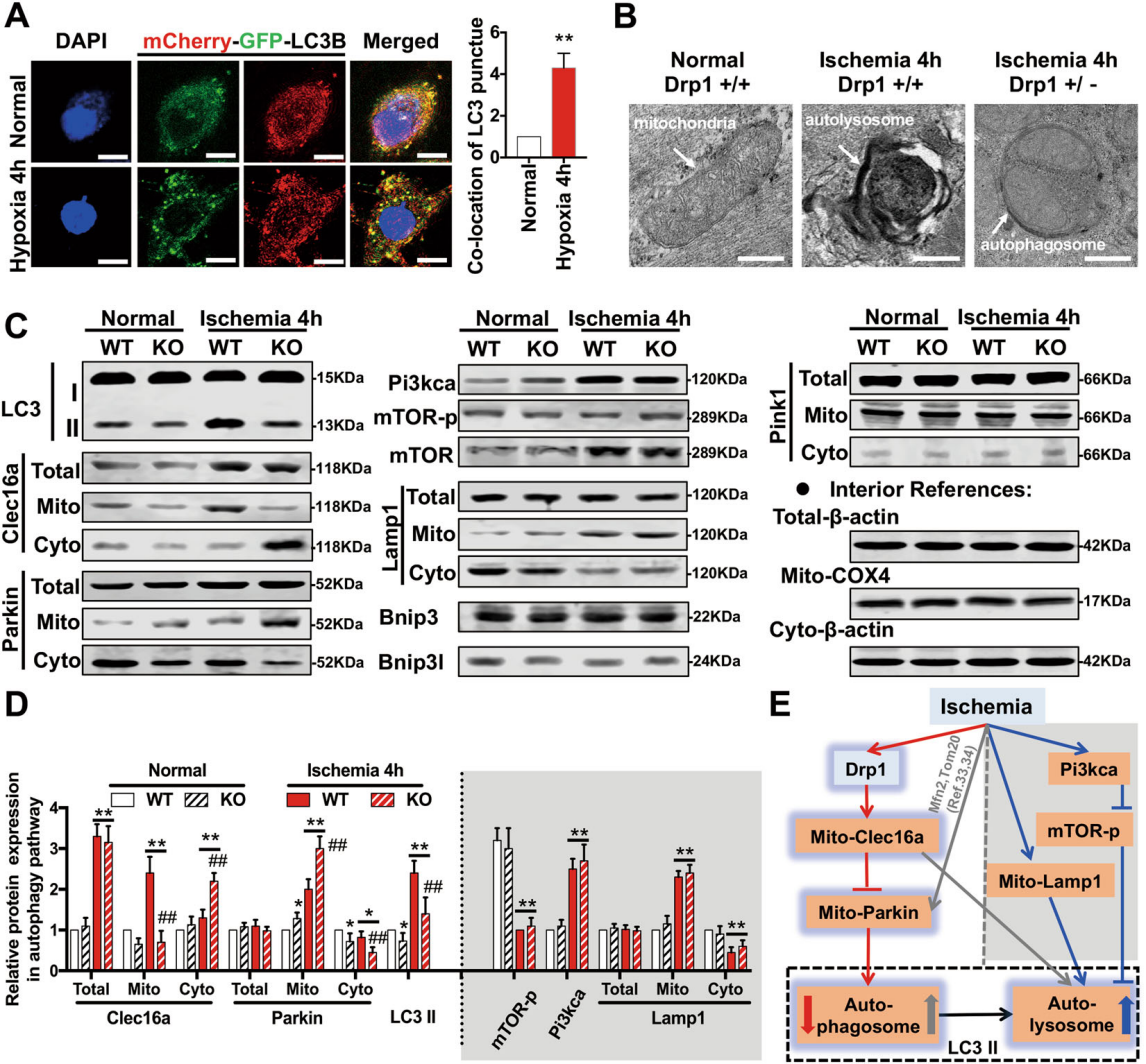
Drp1 participates in autophagy after ischemic injury via the Clec16a-Parkin pathway. **a** Representative confocal images and statistical analysis of VSMCs transfected with mCherry-GFP-LC3B in different groups (63X_bar, 25 μm). **b** TEM images representing autophagy status in different groups (bar, 350 nm). **c** Western blot analysis of autophagy-related protein in WT or Drp1 KO mice after a 4 h ischemic period, with β-actin as internal reference protein for total level and cytoplasm fraction and ANT as internal reference protein for mitochondrial fraction. **d** Statistical analysis of western blot results in Fig. 4C. **e** Schematic diagram of Drp1-mediated autophagy pathways after ischemic injury. *p < 0.05 and **p < 0.01 compared to the WT in normal samples. ^#^*p* < 0.05 and ^##^*p* < 0.01 compared to WT after 4 h ischemic period.

We found that LC3B puncta increased significantly in hypoxia-treated VSMCs (Fig. 4A), indicating the existence of autophagy changes in vascular cells after ischemic injury. However, the specific mechanism is unclear.

Therefore, we used TEM to further assess autophagy in vascular tissues after ischemic injury. We found that, compared to WT mice under normal conditions, autophagy in the vascular tissues of WT mice increased significantly after 4 h ischemia and principally manifested in the form of autolysosomes. Autophagy also increased after 4 h ischemia in Drp1 KO mice but mainly existed in the form of autophagosomes (Fig. 4B). Next, we screened autophagy-related proteins and examined their expression in Drp1 KO mice after ischemia (Fig. 4C). We found that the LC3 II/I ratio increased markedly after ischemia, while Clec16a increased significantly after ischemia, which were both attenuated by Drp1 KO (Fig. 4C). Total Parkin protein expression did not alter after ischemia, but Parkin appeared to be recruited to mitochondria^31^. Moreover, increase in mito-Parkin was detected in Drp1 KO mice (Fig. 4C,D). These results suggest that Drp1 may upregulate mito-Clec16a and inhibit the recruitment of mitochondrial Parkin after ischemic injury, which was closely related to the formation of autophagosomes. Clec16a is a key protein interacting with E3 ubiquitin ligase NRDP1 in regulating autophagic flux. Clec16a also inhibits the formation of autophagosomes mediated by Parkin, but promotes the fusion of autophagosomes and lysosomes to form autolysosomes^32^. Hence, our results suggest that Drp1 regulates autophagy after ischemic injury mainly through mito-Clec16a upregulation, thereby inhibiting the recruitment of Parkin to mitochondria for forming mitochondrial autophagosomes (Fig. 4E).

Moreover, Pi3kca was increased significantly and mTOR was inhibited after ischaemic injury, which was consistent with the results of our previous functional enrichment network (Fig. 2E), suggesting that the autophagy pathway was activated after ischemic injury. However, this process was not related to the level of Drp1 (Pi3kca, mTOR, and mTOR-p expression did not change significantly after Drp1 KO) (Fig. 4C–E). The recruitment of Lamp1, a protein closely related to autolysosome formation, to mitochondria increased significantly after ischemia. However, unlike the formation of autolysosomes mediated by Clec16a, Lamp1-mediated autolysosome formation was Drp1-independent (Fig. 4C–E).

**Fig. 5:**
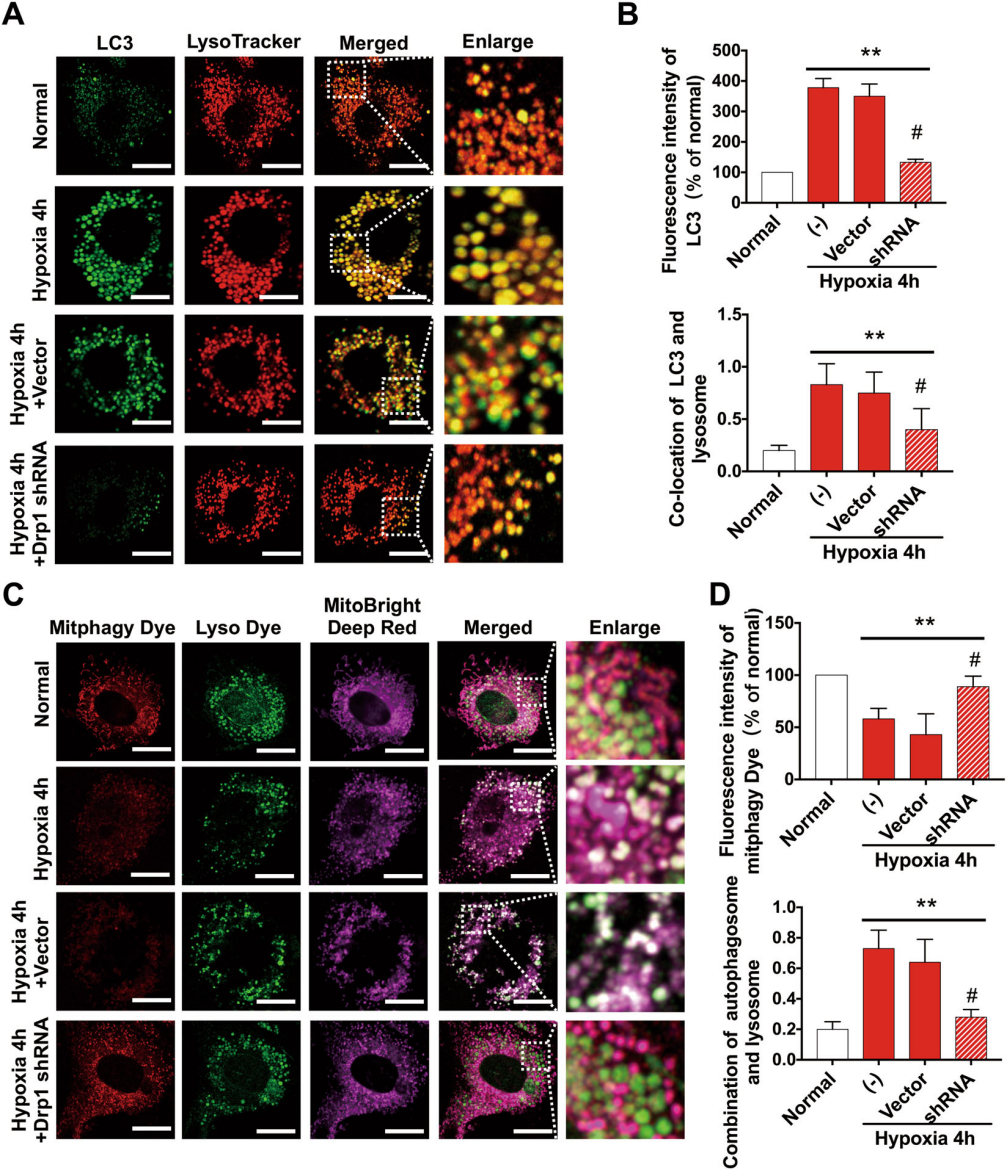
Drp1 affects the formation of autophagosomes after ischemic injury. **a** Representative confocal images of VSMCs transfected with GFP-LC3 and LysoTracker® in different groups (63X_bar, 25 μm). **b** Statistical analysis of colocation results in Fig. 5A. **c** Representative confocal images of mitochondrial autophagosomes and autolysosomes in VSMCs in different groups (63X_bar, 25 μm). **d** Statistical analysis of colocation results in Fig. 5C. *p < 0.05 and **p < 0.01 compared to the normal group. ^#^*p* < 0.05 and ^##^*p* < 0.01 compared to the group subjected to hypoxia for 4 h.

To further validate the effects of Drp1 on autophagic flux after ischemic injury, we applied Drp1 shRNA treatment to deplete about 70% of Drp1 of VSMCs induced by hypoxia at the cellular level (Fig. S3A,B). We found that GFP-LC3 increased markedly after 4 h hypoxia and colocalized with lysosomes labelled by LysoTracker^®^ (Fig. 5A,B), supporting that autophagy (macroautophagy) increased significantly after ischemia, mainly in the form of autolysosomes.

To verify the inhibitory effect of Drp1 on mitophagy after ischemic injury, we stained both mitochondrial autophagosomes and autolysosomes. The results showed that there was a certain degree of mitophagy under normal conditions, but colocalization of autophagosomes and lysosomes was rare. Mitophagy was inhibited after 4 h hypoxia, suggesting an unhealthy accumulation of mitochondria. Colocalization of mitochondrial autophagosomes and lysosomes also increased significantly. After Drp1 shRNA treatment, mitophagy recovered and colocalization of autophagosomes and lysosomes was decreased accordingly (Fig. 5C,D).

### Drp1 participates in apoptosis after ischemic injury by promoting BAX mitochondrial translocation

**Fig. 6:**
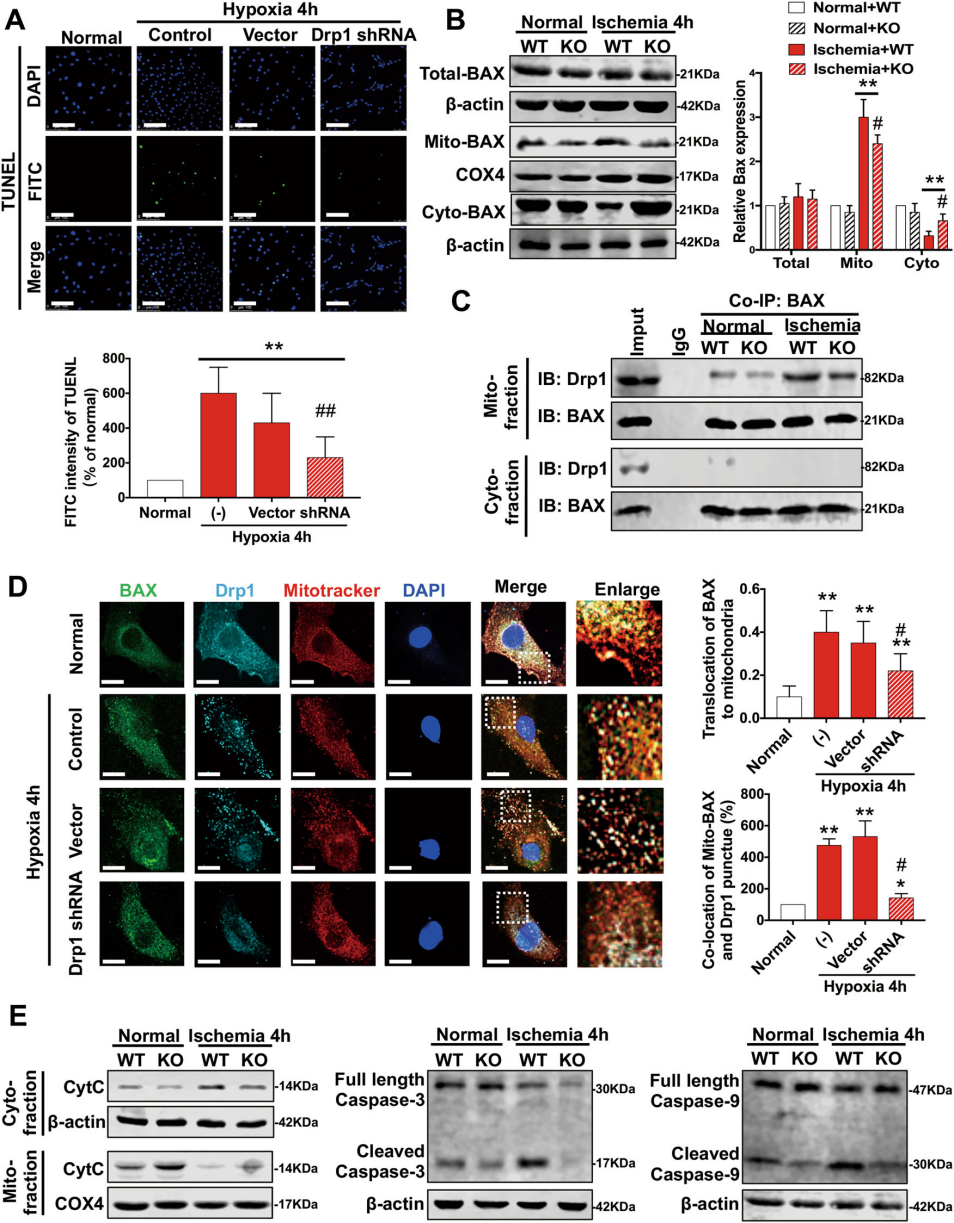
Drp1 participates in apoptosis after ischemic injury by promoting BAX mitochondrial translocation. **a** TUNEL confocal images and statistical analysis of hypoxia-treated VMSCs after Drp1 shRNA (20X_bar, 100 μm). **b** Western blot analysis of BAX expression in different fractions after Drp1 KO. **c** Co-IP results for the combination of Drp1 and BAX in WT or Drp1 KO mice after 4 h ischemic period. **d** Representative confocal images and statistical analysis of colocalization between BAX, Drp1, and mitochondria in different groups. **e** Western blot analysis of the downstream proteins of apoptosis in WT or Drp1 KO mice after 4 h ischemic period. *p < 0.05 and **p < 0.01 compared to the normal group in vitro or WT in vivo. ^#^*p* < 0.05 and ^##^*p* < 0.01 compared to the group subjected to hypoxia for 4 h in vitro or WT after ischemia in vivo.

The FITC value of the TUNEL index decreased by 62% after intervention with Drp1 shRNA (Fig. 6A), confirming that Drp1 plays an important role in apoptosis after ischemia. The total protein expression of BAX had no significant change after ischemia but mito-BAX clearly increased and cyto-BAX clearly decreased, suggesting BAX mitochondrial translocation. Furthermore, this process could be reversed by Drp1 KO (Fig. 6B).

It has been reported that ischemia may also cause the activation of Drp1 as well as its translocation to mitochondria^16^. Thus, we performed Co-IP of Drp1 and BAX and found that they can combine on mitochondria in vascular tissues after ischemic injury, while there was no relativity between Drp1 and BAX in cytoplasmic components (Fig. 6C). At the same time, we found that the recruitment of mito-Drp1 to BAX and the translocation of BAX to mitochondria could be inhibited after Drp1 KO (Fig. 6C).

To further confirm the inhibitory effect of Drp1 intervention on ischemia-induced BAX mitochondrial translocation, we observed the colocalization of BAX, Drp1, and mitochondria in VSMCs after 4 h hypoxia. Fluorescent images showed that the colocalization of the BAX-Drp1 complex to mitochondria after ischemia was about four times higher than under normal conditions, which was reversed by 70% after treatment with Drp1 shRNA (Fig. 6D).

Next, we studied the downstream pathways of apoptosis and found that CytC release increased markedly and caspase-3 and caspase-9 were activated after ischemic injury (Fig. 6E), which was consistent with previous studies on hypoxic-ischemic brain damage^33^. These downstream pathways of apoptosis could also be reversed by Drp1 KO, further verifying the facilitation of Drp1 on the apoptotic process after ischemic injury.

### Drp1 participates in cellular metabolic disorders after ischemic injury by inhibiting GSH elimination to free radicals

**Fig. 7:**
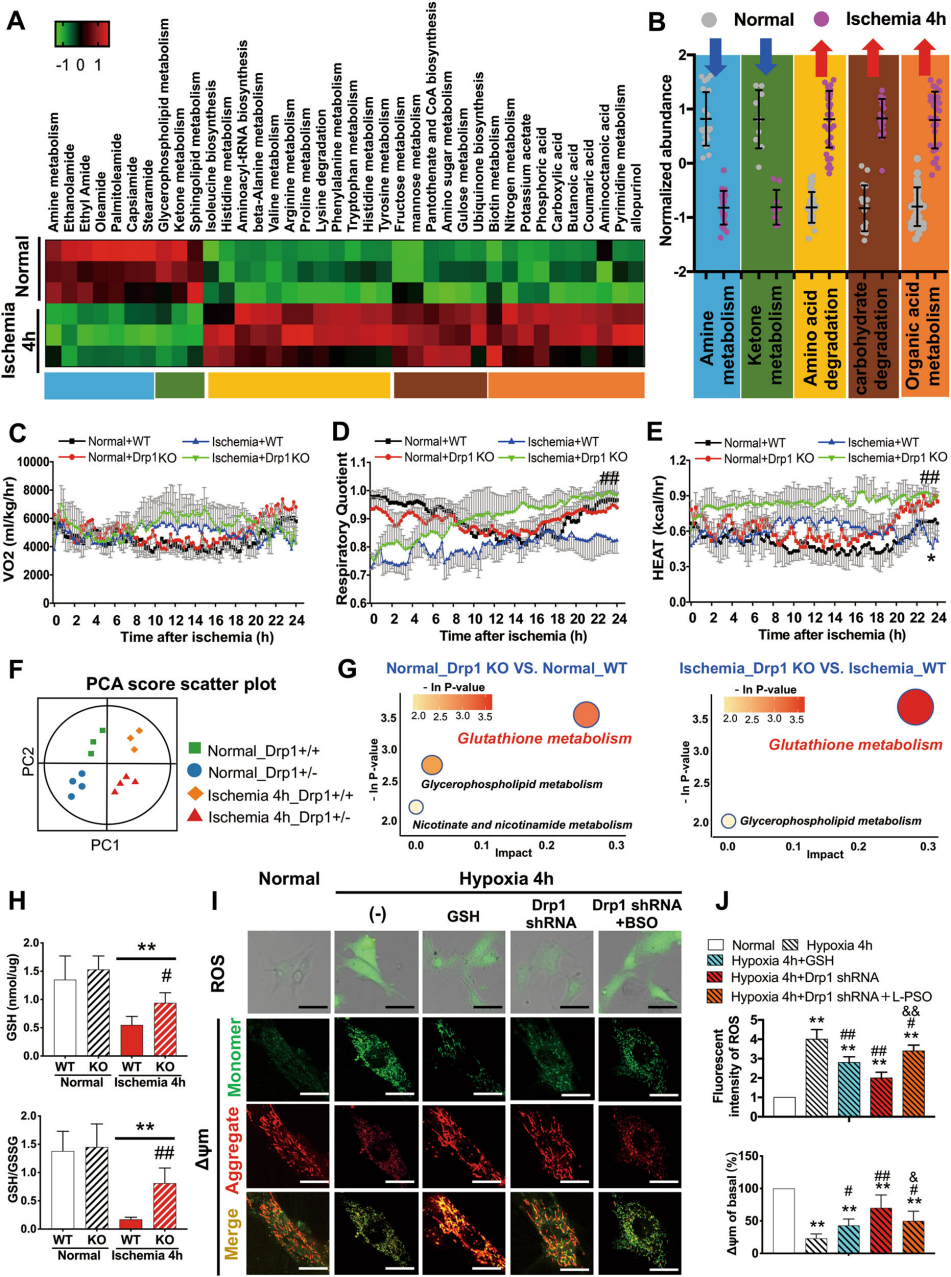
Drp1 participates in disordered cellular metabolism after ischemic injury by inhibiting GSH elimination of free radicals. **a** Metabonomics heatmap in vascular tissue after 4 h ischemic period. **b** Metabolic classification of metabolic molecules and pathways represented in Fig. 7A. The background colors correspond to the color bars in Fig. 7A. **c** 24-h metabolic cage detection to monitor oxygen consumption (VO2) of Drp1 KO mice after ischemia. **d** 24-h metabolic cage detection to monitor respiratory quotient of Drp1 KO mice after ischemia. **e** 24-h metabolic cage detection to monitor energy metabolism of Drp1 KO mice after ischemia. **f** Principal component analysis (PCA) indicated substantial diversity among the four groups and all samples were within the 95% confidence interval (Hotelling’s T-squared ellipse). **g** Bubble diagram of metabolic pathway enrichment after Drp1 KO under normal and ischemic conditions. **h** Mitochondrial glutathione (GSH) and oxidized glutathione (GSSG) levels in different groups. **i** Representative confocal images of ROS and △Ψm after intervening Drp1 and GSH in hypoxia-treated VSMCs. **j** Statistical analysis of Fig. 7G. *p < 0.05 and **p < 0.01 compared to the normal group in vitro or WT in vivo. ^#^*p* < 0.05 and ^##^*p* < 0.01 compared to the group subjected to hypoxia for 4 h in vitro or WT after ischemia in vivo.

Previous studies have shown that there are obvious cellular metabolic disorders after ischemia induced by cancer or brain damage^34^. Therefore, we quantitatively analysed 400 metabolic markers and found that a series of metabolic molecules in vascular tissue varied significantly after ischemic injury (Fig. 7A). We classified the metabolic pathways of these metabolic molecules and found that (1) amine metabolism and (2) ketone metabolism significantly after ischemia (Fig. 7B), which was consistent with our previous transcriptome findings on metabolic changes after ischemic injury in vascular tissues (Fig. 1A). Other markedly changed metabolic pathways in this process included (3) amino acid degradation, (4) carbohydrate degradation, and organic acid metabolism (Fig. 7B). The differential metabolites exhibiting significant changes after ischemic injury are shown in Fig. 7A in detail. Our metabonomic results illuminated the specific metabolic pathways that underwent significant changes in vascular tissues after ischemic injury.

We then performed 24-h metabolic cage detection to monitor metabolic changes in WT and Drp1 KO mice under normal and ischemic conditions, and found that Drp1 KO had no effects on oxygen consumption (VO2) (Fig. 7C) but respiratory quotient of Drp1 KO mice increased significantly after ischemia compared with WT mice (Fig. 7D). In addition, we also found that the energy metabolism level of Drp1 KO mice was slightly higher than that of WT mice (*p* < 0.05), and the difference in energy metabolism between WT and Drp1 KO mice was more significant after ischemia (*p* < 0.01) (Fig. 7E).

To explore the regulatory mechanism of Drp1 in metabolic disorders after ischemic injury in vascular tissues, we extracted SMAs from Drp1 KO mice for metabonomic analysis to determine the metabolic molecules and pathways related to Drp1 both under normal and ischemic conditions (Figs. 7F and S4A–D, Table 2). Our data suggest that Drp1 may have a significant inhibitory effect on glutathione under both normal and ischemic conditions (Fig. S4,C,D). The results revealed by the bubble diagram of metabolic pathway analysis also verified that the glutathione metabolism pathway was highly enriched after Drp1 KO (Fig. 7G).

**Table 2:** Differentially expressed metabolites in Drp1 KO mice under normal or ischemia conditions.

Groups	Metabolite name	VIP	*P*-Value	Fold-change
Normal_Drp1 KO VS. Normal_WT	C17-Sphinganine	2.0046	0.0050	0.6062
	Lauryl diethanolamide	1.7902	0.0286	1.8109
	12-amino-octadecanoic acid	1.3755	0.0473	0.4462
	Triphenylphosphine oxide	1.7559	0.0452	1.5391
	4-oxo-2-Nonenal	2.1491	0.0225	1.7825
	lL-Proline	1.6090	0.0422	1.5644
	Nicotinamide	1.6888	0.0443	1.5412
	Glutathione	1.2241	0.0495	1.4872
Ischemia_Drp1 KO VS. Ischemia_WT	Met His Lys	1.8094	0.0222	1.1405
	Glutathione	1.5762	0.0422	1.2040
	4-Hydroxypenbutolol	1.7178	0.0477	1.3998
	Lauryl diethanolamide	2.2267	0.0038	1.7960
	Adenosine 3’-monophosphate	1.9768	0.0071	0.4543
	Met Ser Asp Thr	1.7272	0.0351	1.1581
	Lys Cys His	2.1264	0.0009	1.4878
	N-methyl arachidonoyl amine	1.7289	0.0351	0.7240
	Dihydroxygrosheimin	1.6348	0.0494	1.1153

The mitochondrial glutathione (GSH) content and oxidized glutathione (GSSG) level were assessed, and both showed significant decreases in vascular tissues after ischemia 4 h; however, the decreases were attenuated after Drp1 KO (Fig. 7H). Next, we assessed ROS and △Ψm levels after hypoxia by intervening with Drp1 and GSH, respectively (Fig. 7I). The results showed the increase in ROS and decrease in △Ψm could be inhibited by supplementing with GSH (10 mM) after hypoxia^35^. However, the effect of Drp1 shRNA treatment on mitochondrial function was more pronounced after hypoxia. We also used GSH inhibitor buthionine sulfoxamine (BSO) (1 mM)^35,36^ on the basis of Drp1 shRNA treatment and found that the ROS level increased by 28% and △Ψm decreased by 20% (Fig. 7I–J).

**Fig. 8:**
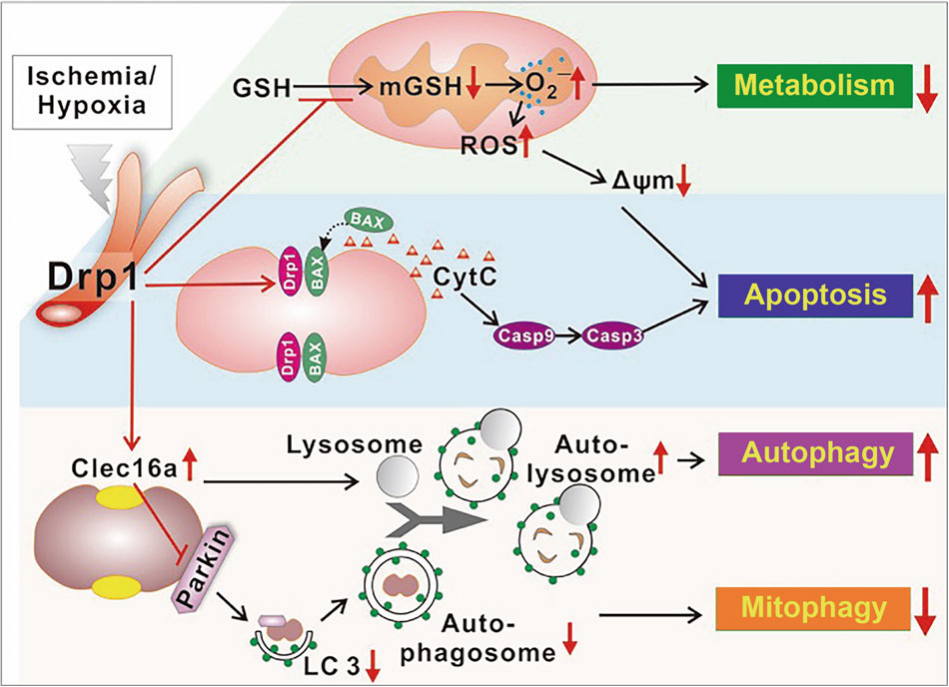
Schematic diagram for the mechanisms of Drp1-regulated autophagic, apoptotic, and metabolic pathways under ischemic injury. Drp1 plays an important role in the regulation of autophagy, apoptosis, and metabolism after ischemic injury. (i) Drp1 may inhibit mitophagy by upregulating mito-Clec16a, inhibiting mito-Parkin recruitment and thus affecting the formation of autophagosomes in vascular tissues after ischemic injury. Likewise, other Drp1-independent pathways in autophagy upregulation may be implicated in this process. (ii) ischemia-induced Drp1 activation may facilitate apoptosis by inducing BAX translocation to mitochondria and thereby increasing Cytochrome C release and caspase-3/-9 activation. (iii) Drp1 may lead to disordered metabolism and inhibit mitochondrial GSH level after ischemic injury, thereby impairing free radical scavenging, leading to further increases in ROS, △Ψm, and aggravation of mitochondrial dysfunction after ischemic injury.

In summary, the results of metabonomic analysis and basic experimental evidence revealed that Drp1 can inhibit mitochondrial GSH levels after ischemic injury, thereby impacting free radical scavenging, leading to a further increase in ROS, further decrease in membrane potential, and further aggravation of mitochondrial dysfunction after ischemic injury (Fig. 8).

## Discussion

In this study, we showed a role of mitochondrial pathways in the early stage of ischemia. We also found that mitochondrial dysfunction occurred in the late stage of ischemia. Our data showed that Drp1 might participate in the following biological processes: (1) Most mitochondrial pathways are regulated by Drp1 after ischemia. (2) The decrease in actin filament aggregation and the increase in microtubule mobility after ischemia could be reversed by Drp1 knockout. These processes may be regulated by Drp1-mediated cytoskeleton remodelling and binding of related protein complexes. (3) Ischemia-induced CytC release, NO homeostasis imbalance and intracellular pH disorder may be regulated by Drp1. (4) Changes in peroxisomes and lysosomes after ischemia may also be associated with the mitochondrial pathways regulated by Drp1.

Autophagy alters after ischemic injury^37–40^, while some suggested that autophagy is a protective mechanism against ischemia, others showed that the protective effects are limited at early stage of ischemia^41–43^. Studies on cerebral ischemia^44^ found that both autophagy and mitophagy were activated in the early stage, but further studies of mitophagy changes in the late stage of ischemia are unavailable. It was also shown that autophagy could be activated during ischemia and reperfusion, but autophagy was harmful during ischemia and conducive to neuroprotection during reperfusion^45^. Research on myocardial ischemia showed that autophagy was protective during ischemia, but harmful during reperfusion^46^. These contradictory reports may be related to differences in ischemia models, cell types, and signalling pathways involved.

In our study, we also found that both autophagy and mitophagy were activated after ischemic injury in vascular tissues. However, we found that mitophagy exhibited a Drp1-dependent-inhibitory regulatory mechanism during this process. Drp1 inhibited mitophagy under ischemic conditions by upregulating mito-Clec16a, inhibiting mito-Parkin recruitment and thus affecting the formation of the autophagosome. It was shown that the interaction between Clec16a and NRDP1 of E3 ubiquitin ligase not only inhibits the generation of autophagosomes mediated by Parkin, but also promotes the maturity of autophagosomes and the formation of autolysosomes^32^, which was consistent with our report. Moreover, the “inconsistency between autophagy and mitophagy” reflected in recent reports was consistent with our observation in autophagy and mitophagy after ischemia in VSMCs^47^. At the same time, the “increase in autolysosomes after ischemic injury” that we observed with an electron microscope also supported the evidence that ischemic injury could promote the maturation process of autophagosomes, and that this change may be related to the formation of autolysosomes promoted by the upregulation of Lamp1 after ischemic injury.

Recent studies have shown that autophagy could interact with apoptosis and necrosis signalling pathways and even regulate apoptosis^48^. The absence of Atg7 in autophagy could reduce caspase-3 activation and neuronal death induced by ischemia^49^. The increase in lysosomal hydrolase induced by autophagy could participate in the process of apoptosis by activating caspase-3^50^. Nevertheless, studies have proposed that autophagy may have an inhibitory effect on apoptosis after ischemia^43,51^. It was reported that rapamycin, an inducer of autophagy, could inhibit the process of apoptosis by preventing the translocation of BAX and Bad to mitochondria^43^. The close relationship between autophagy and apoptosis after ischemic injury was also verified in the vascular endothelium^52^, gliacytes^53^, and other cell types, but these results are also controversial.

Our research showed that apoptosis was upregulated in VSMCs after ischemic injury and that this process is closely related to the Drp1-BAX pathway. Our results verified that the changes in Drp1 modification in vascular tissues could promote the recruitment of BAX to mitochondria, Cytochrome C release, and caspase activation, which was also verified at the cellular level. Additionally, Drp1 could interact with BAX directly to induce the translocation of BAX to mitochondria in osteosarcoma^54^, which was consistent with our data.

Studies have shown that disordered metabolism after ischemia can result in severe tissue hypoxia and vascular dysfunction^55^. Recent research indicates that, in ischemic injury, there is a significant difference between metabolic changes leading to reduced systemic perfusion when compared to metabolic changes in local ischemia or reperfusion injury of a single organ^56^. Dysfunction of the vascular endothelium cell barrier and the viability of adenylate cyclase are related to vascular leakiness caused by a reduction in cAMP levels^57,58^. In addition, ischemic injury could inhibit the viability of prolyl hydroxylase and thus activate the transcription of the hypoxic-inflammatory signalling mediators Hif1a and NF-κB^59^. Here, we performed transcriptomic and metabolomic analyses to demonstrate that amine metabolism and ketone metabolism in vascular tissues are downregulated significantly after ischemic injury. Moreover, metabolomic results determined that the degradation pathways of amino acids and carbohydrates in vascular smooth muscle tissues are clearly upregulated.

It has been shown that energy metabolism alters from fatty acid oxidation to glycolysis with higher oxidation efficiency during ischemic injury^60^. The mitochondria, as important sources of cellular energy, play a crucial role in energy metabolism during ischemic injury. Activation of mitochondrial aldehyde dehydrogenase 2 was closely related to disturbances in energy metabolism after ischemia^61^. The screening results of differential metabolites in this study showed that the capacity of VSMC glutathione to clear free radicals was compromised after ischemic injury, consisting with preliminary findings on cerebral ischemia^62^. The mitochondrial respiratory chain—an important process for the generation of oxygen radicals in vivo—can be inhibited by the reduction of mitochondrial membrane potential and other factors, leading to an increase in free radicals, lipid peroxidation of mitochondrial membranes, mtDNA damage, and deterioration of mitochondrial dysfunction, thus inducing a reinforcing feedback loop. GSH can greatly increase the content of combined enzyme agent I and thus inhibit this feedback loop by preventing free radicals from generating chains. Here we showed that this process was closely associated with the modification of Drp1 expression, the mitotic protein of mitochondria, and that the mitochondrial dysfunction mediated by Drp1 after ischemic injury may also be related to the upregulation of ROS levels and the reduction of △Ψm caused by inhibiting the capacity of glutathione to clear free radicals.

In conclusion, our study reveals that the molecular pathways linked to mitochondria change with the duration of ischemia. Mitochondrial morphology and dynamics are mainly involved in the early stages of ischemia, while mitochondrial dysfunction is mainly involved in the later stages of ischemia. Drp1 deficiency illustrates the important role of Drp1 in ischemia-induced autophagy, apoptosis, and metabolic pathways through multiple fission-independent mechanisms. It would be interesting to delineate the exact mechanisms of Drp1-regulated mitochondrial pathways in different phases of ischemic injury, in the hopes of developing therapies for ischemic injury that target Drp1-mediated regulation of mitochondrial homeostasis.

## Supplementary information

Supplementary figure legends

Figure S1

Figure S2

Figure S3

Figure S4
