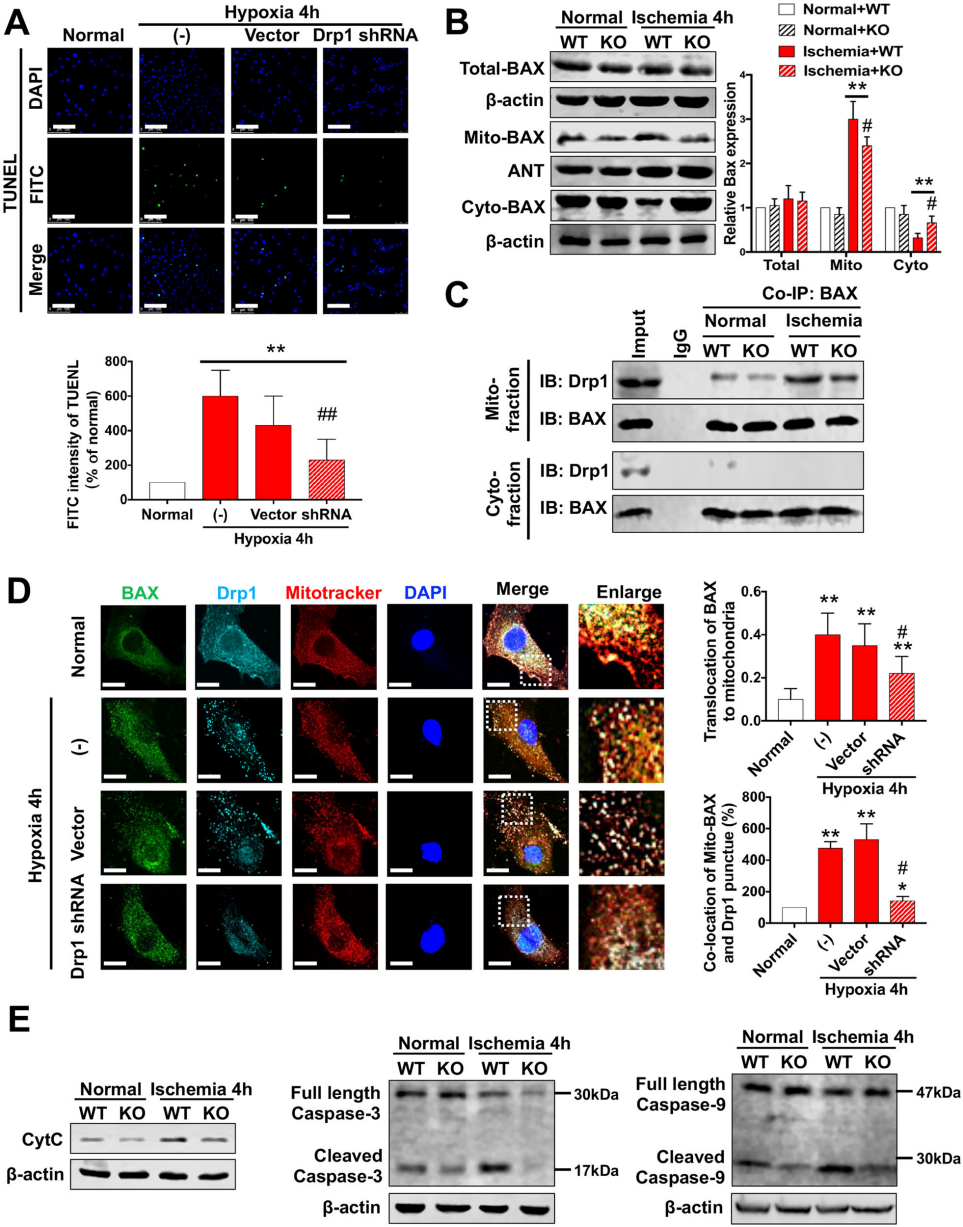# Correction: Drp1 regulates mitochondrial dysfunction and dysregulated metabolism in ischemic injury via Clec16a-, BAX-, and GSH- pathways

**DOI:** 10.1038/s41419-024-07314-0

**Published:** 2025-01-13

**Authors:** Chenyang Duan, Lei Kuang, Xinming Xiang, Jie Zhang, Yu Zhu, Yue Wu, Qingguang Yan, Liangming Liu, Tao Li

**Affiliations:** State Key Laboratory of Trauma, Burns and Combined Injury, Second Department of Research Institute of Surgery, Daping Hospital, Army Medical University, 400042 Chongqing, PR China

Correction to: *Cell Death & Disease* 10.1038/s41419-020-2461-9, published online 20 April 2020

In Figure 6E, the Western Blot band for the β-actin loading control in the far-right lane was unintentionally misused. We have corrected this and provided the revised Figure 6 below. As the expression of the target proteins was already normalized using total protein quantification, this adjustment does not impact the scientific conclusions or figure legends of the article.

Original Figure 6
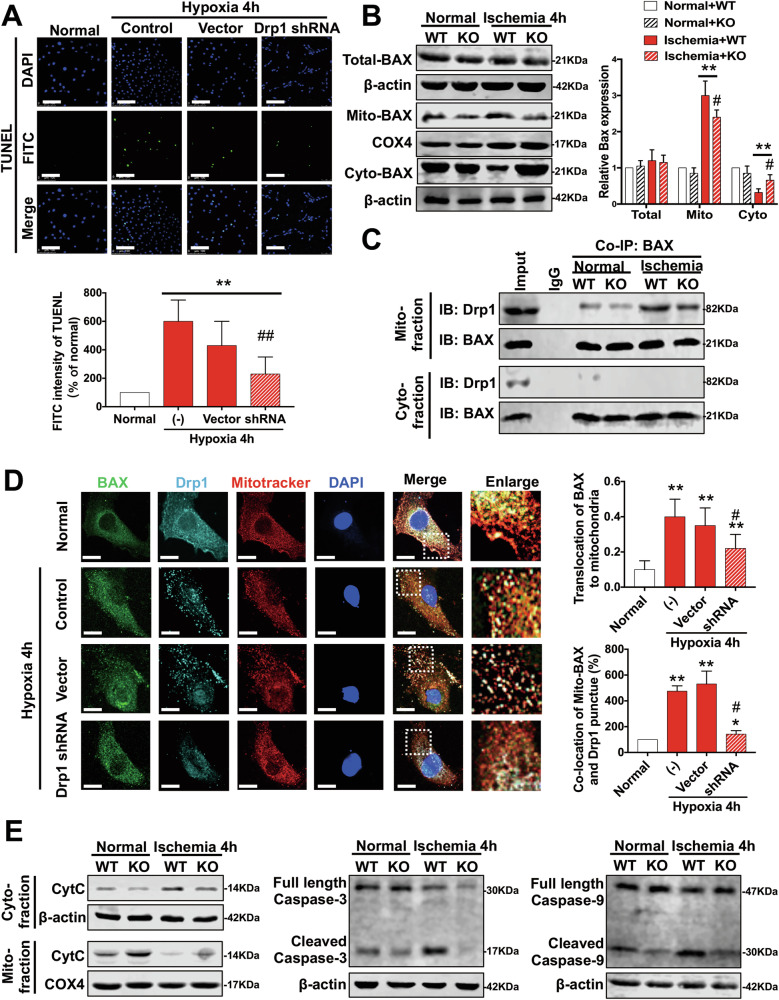


Revised Figure 6