# Supplementary material for: Drp1 regulates mitochondrial dysfunction and dysregulated metabolism in ischemic injury via Clec16a-, BAX-, and GSH- pathways
Source: Cell Death Dis. 2020 Apr 20;11(4):251. doi: 10.1038/s41419-020-2461-9 (PMC7170874; doi:10.1038/s41419-020-2461-9)
Supplement: Supplementary file 1 — Supplementary figure legends [file 41419_2020_2461_MOESM1_ESM.docx]

**Supplementary figure legends**

**Figure S1. The establishment of Drp1 KO mice and demonstration of knockout effect.** (A) The strategy for Drp1 KO mice (Drp1+/-). (B) PCR identification results for Drp1 KO mice. (C) Sequencing results to show reduced gene read value of exon 2 in Drp1 KO mice. (D) Western blot analysis to confirm the Drp1 knockout effect at the protein level. (E) Immunofluorescence images of Drp1 expression in SMAs after Drp1 KO (20X_bar, 100 μm). * p < 0.05 and ** p < 0.01 compared with WT in normal. # p < 0.05 and ## p < 0.01 compared with WT after 4 h ischemic period.

**Figure S2. Trend classification for series-cluster analysis of Drp1-mediated biological pathways under normal and ischemic conditions.** (A) The trend classification for series-cluster analysis under normal conditions. (B) The trend classification for series-cluster analysis under ischemic conditions. “0-7” represent eight types of trend. “0”: Pathways which were down-regulated in “Normal_Drp1+/-” or “Ischemia 4h _Drp1+/+” groups and were further down-regulated in the “Ischemia 4h_Drp1+/-” group; “1”: Pathways which were down-regulated in “Normal_Drp1+/-” or “Ischemia 4h _Drp1+/+” groups but were not further changed in the “Ischemia 4h_Drp1+/-” group; “2”: Pathways which were down-regulated in “Normal_Drp1+/-” or “Ischemia 4h _Drp1+/+” groups but were recovered in the “Ischemia 4h_Drp1+/-” group; “3”: Pathways which were not changed in “Normal_Drp1+/-” or “Ischemia 4h _Drp1+/+” groups but were down-regulated in the “Ischemia 4h_Drp1+/-” group; “4”: Pathways which were not changed in “Normal_Drp1+/-” or “Ischemia 4h _Drp1+/+” groups but were up-regulated in the “Ischemia 4h_Drp1+/-” group; “5”: Pathways which were up-regulated in “Normal_Drp1+/-” or “Ischemia 4h _Drp1+/+” groups but were down-regulated in the “Ischemia 4h_Drp1+/-” group; “6”: Pathways which were up-regulated in “Normal_Drp1+/-” or “Ischemia 4h _Drp1+/+” groups but were not changed in the “Ischemia 4h_Drp1+/-” group; “7”: Pathways which were up-regulated in “Normal_Drp1+/-” or “Ischemia 4h _Drp1+/+” groups and were further up-regulated in the “Ischemia 4h_Drp1+/-” group.

**Figure S3. Demonstration of interfering effects of VSMCs transfected with Drp1 shRNA.** (A) The transfection effects of GFP-Drp1 shRNA on VSMCs determined by fluorescent and optical scope (20X_bar, 75 μm). (B) The interfering effect of Drp1 shRNA on Drp1 expression determined by western blot. * *p* < 0.05 and ** *p* < 0.01 compared to the normal group.

**Figure S4. OPLS-DA permutation test and volcano plot of differentially expressed metabolites after Drp1 KO under normal and ischemic conditions.** (A) OPLS-DA permutation test to assess the favorable stability of the sample data under normal conditions. (B) OPLS-DA permutation test to assess the favorable stability of the sample data under ischemic conditions. (C) Volcano plot of differentially expressed metabolites after Drp1 KO under normal conditions. (D) Volcano plot of differentially expressed metabolites after Drp1 KO under ischemic conditions.
