# Supplementary figures and images for: Drp1 regulates mitochondrial dysfunction and dysregulated metabolism in ischemic injury via Clec16a-, BAX-, and GSH- pathways
Source: Cell Death Dis. 2020 Apr 20;11(4):251. doi: 10.1038/s41419-020-2461-9 (PMC7170874; doi:10.1038/s41419-020-2461-9)

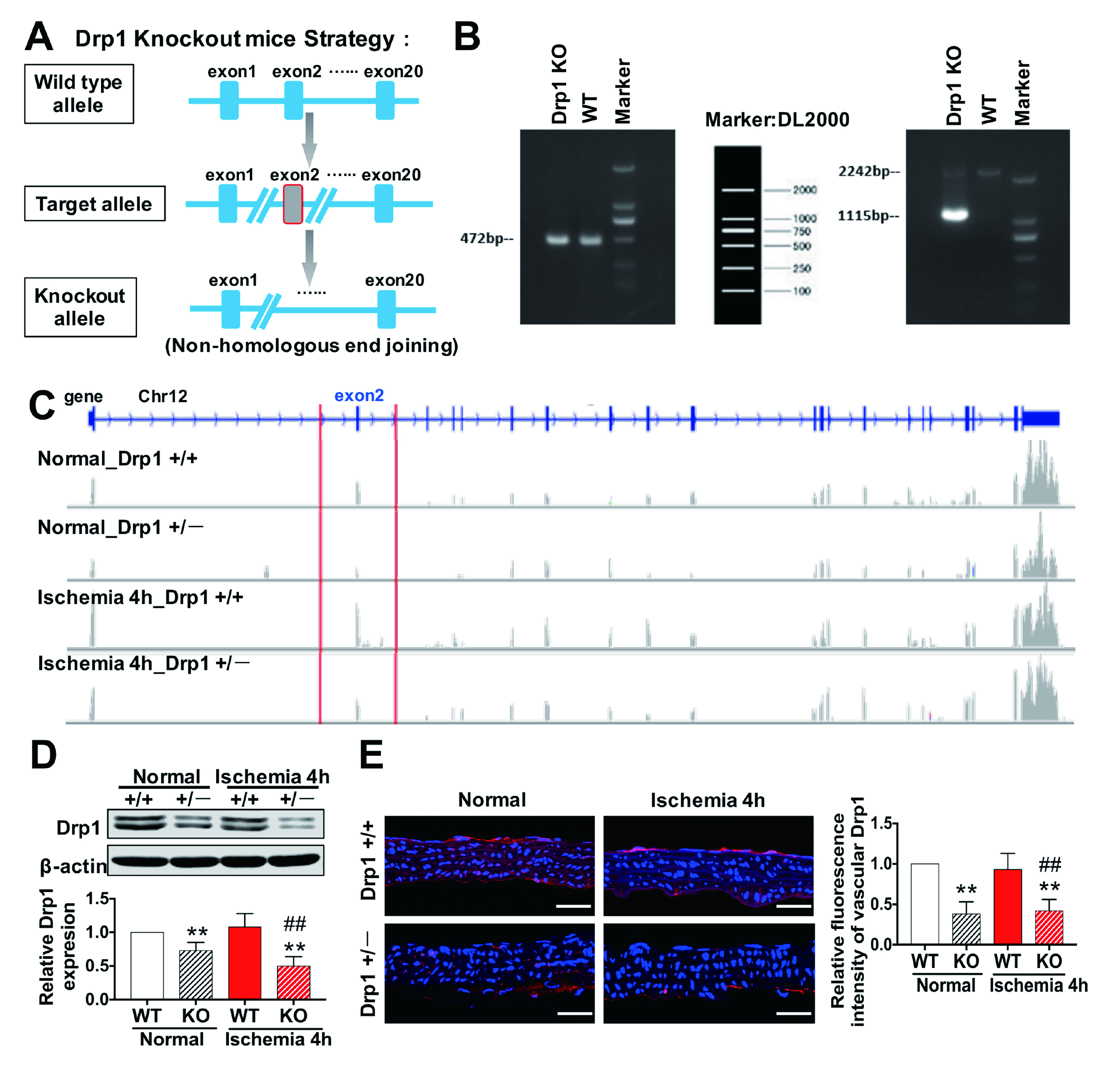

Supplement: Supplementary file 2 — Figure S1 [file 41419_2020_2461_MOESM2_ESM.tif]

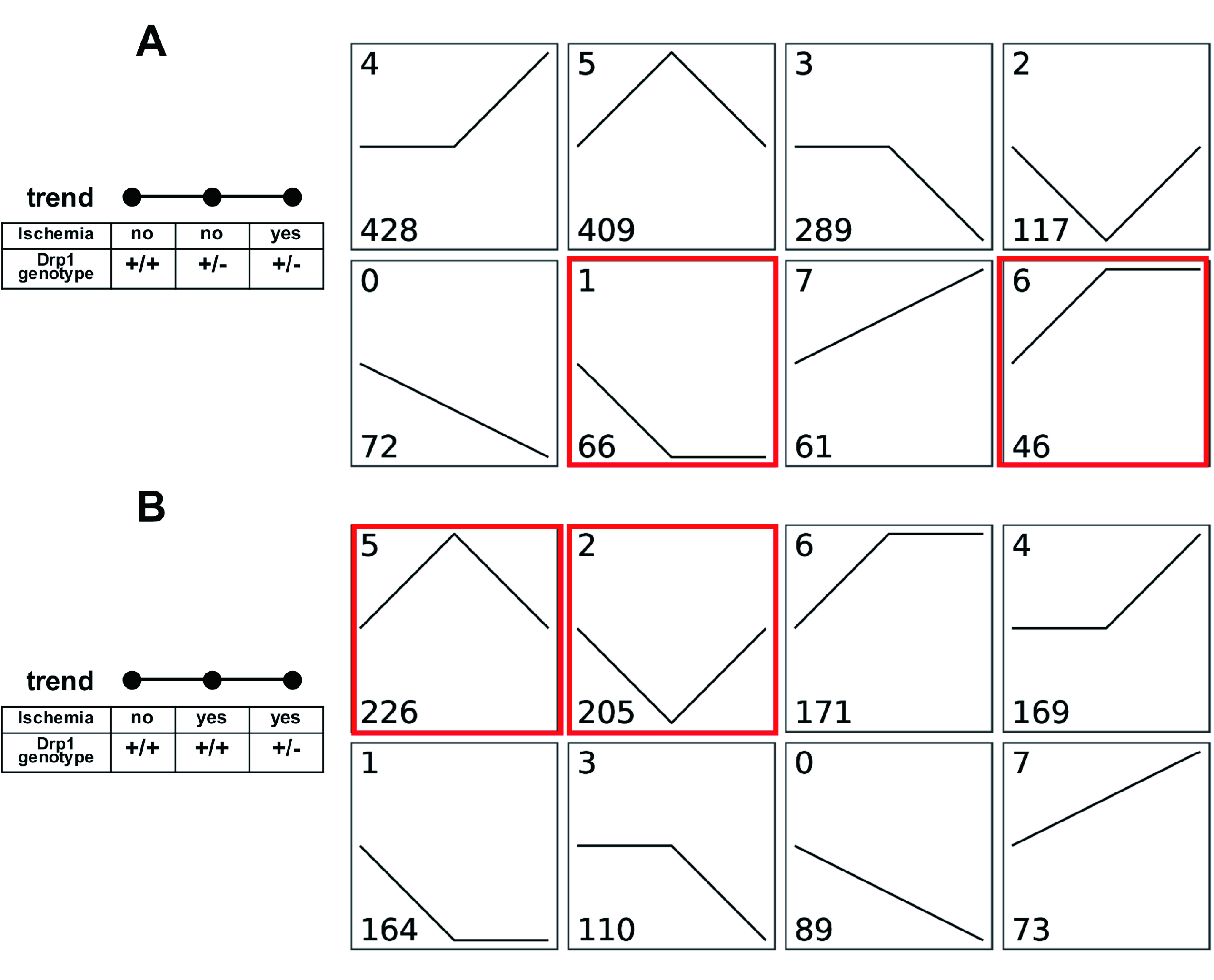

Supplement: Supplementary file 3 — Figure S2 [file 41419_2020_2461_MOESM3_ESM.tif]

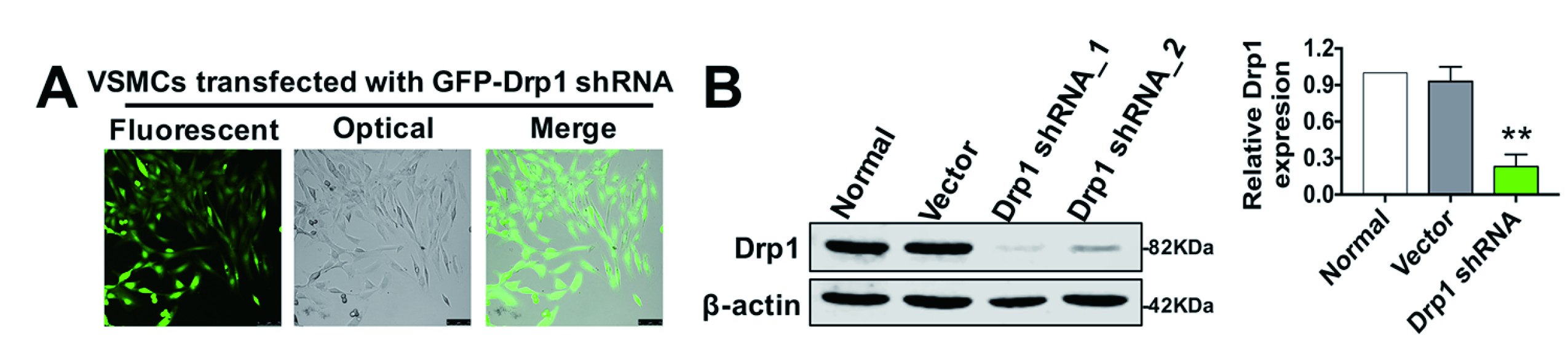

Supplement: Supplementary file 4 — Figure S3 [file 41419_2020_2461_MOESM4_ESM.tif]

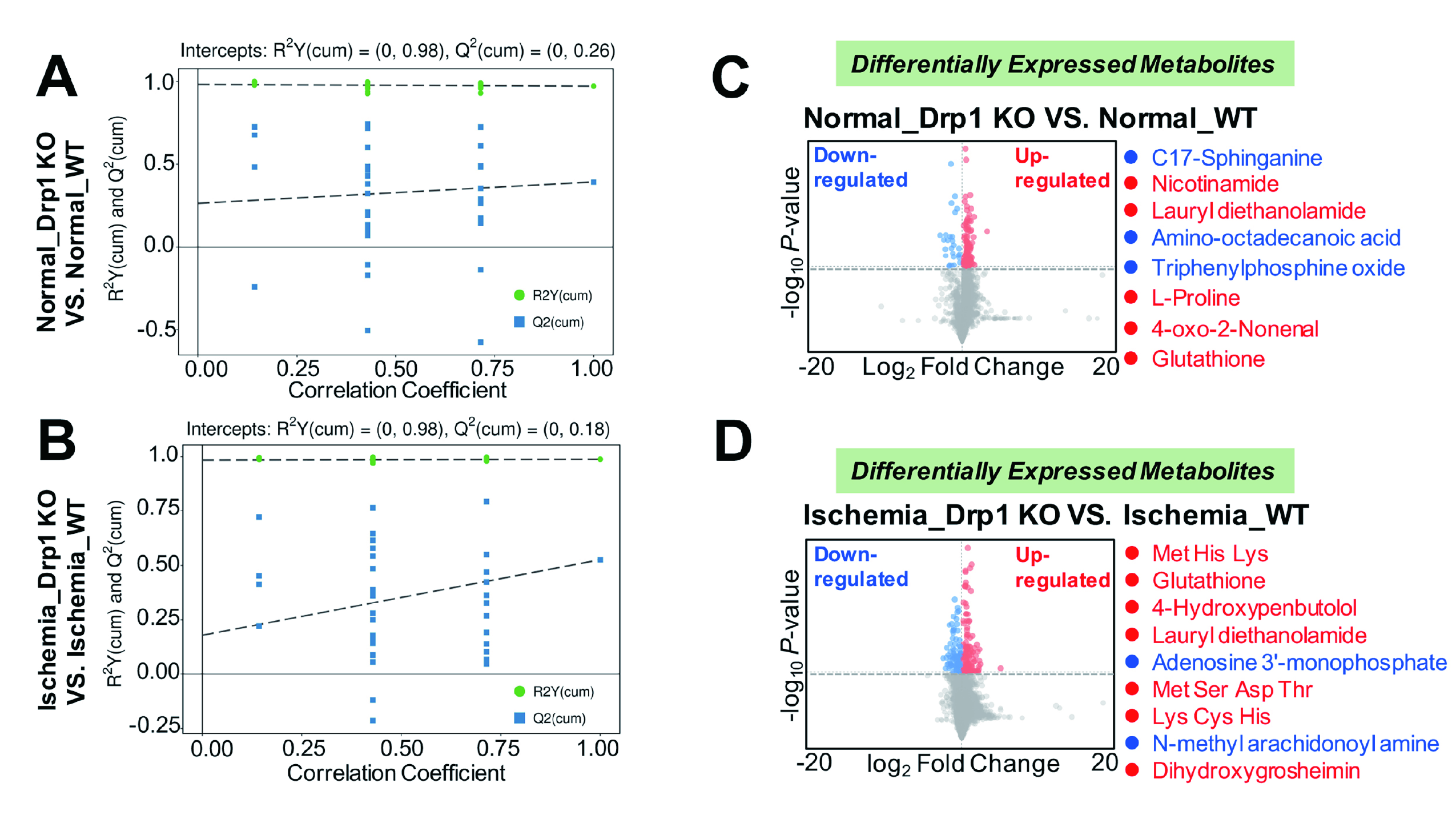

Supplement: Supplementary file 5 — Figure S4 [file 41419_2020_2461_MOESM5_ESM.tif]
